# Role of adenosine A2a receptor in cancers and autoimmune diseases

**DOI:** 10.1002/iid3.826

**Published:** 2023-04-12

**Authors:** Hongling Ye, Junqi Zhao, Xuejing Xu, Dagan Zhang, Han Shen, Sen Wang

**Affiliations:** ^1^ Department of Clinical Laboratory Medicine Nanjing Drum Tower Hospital, Medical School of Nanjing University Nanjing Jiangsu P.R. China

**Keywords:** A2AR, adenosine receptors, autoimmune disease, cancer

## Abstract

Adenosine receptors are P1 class of purinergic receptors that belong to G protein‐coupled receptors. There are 4 subtypes of adenosine receptors, namely A1, A2A, A2B, and A3. A2AR has a high affinity for the ligand adenosine. Under pathological conditions or external stimuli, ATP is sequentially hydrolyzed to adenosine by CD39 and CD73. The combination of adenosine and A2AR can increase the concentration of cAMP and activate a series of downstream signaling pathways, and further playing the role of immunosuppression and promotion of tumor invasion. A2AR is expressed to some extent on various immune cells, where it is abnormally expressed on immune cells in cancers and autoimmune diseases. A2AR expression also correlates with disease progression. Inhibitors and agonists of A2AR may be potential new strategies for treatment of cancers and autoimmune diseases. We herein briefly reviewed the expression and distribution of A2AR, adenosine/A2AR signaling pathway, expression, and potential as a therapeutic target.

## Graphical abstract

A2AR is expressed to some extent on various immune cells, where it is abnormally expressed on immune cells in cancers and autoimmune diseases. This review summarizes the expression and distribution of A2AR, adenosine/A2AR signaling pathway, expression, and potential as a therapeutic target.

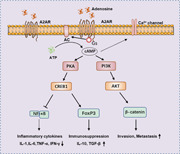

## 1 INTRODUCTION

Adenosine receptors are type receptors Purinergic1 type receptors belonging to G protein‐coupled receptors, which are widely distributed in human tissues, but have different expression levels in different organs, tissues and cells.^1^ Adenosine receptors include four subtypes, A1R, A2AR, A2BR, and A3R.^2^ Adenosine receptors can bind to extracellular adenosine and exert corresponding functions. Adenosine‐induced immunosuppression is mainly related to A2AR and A2BR. For adenosine, A2AR is a high‐affinity receptor, while A2BR is a low‐affinity receptor.^3^, ^4^ A2AR play an immunosuppressive role and have been recognized for a long time. Research by Dr. Ohta and Dr. Sitkovsky has shown that, the A2AR has nonredundant functions in suppressing inflammation and reducing tissue damage, and has suggested that regulation of adenosine/A2AR signaling pathway could be a strategy for tumor therapy.^5^, ^6^ Recent studies have shown that the A2AR is involved in tumor immune escape and is a new immune checkpoint molecule.^7^, ^8^ It also plays an important role in the occurrence and development of autoimmune diseases.^9^, ^10^ In this review, we searched Pubmed and Web of science databases for relevant published literature up to August 2022 using A2AR as the keyword, and screened out the literature related to autoimmune diseases and cancer for review. We focus on the structure and distribution of A2AR, adenosine/A2AR signaling pathway and its regulation of immune cells. This review also summarizes the expression and important roles of A2AR in cancers and autoimmune diseases.

## 2 STRUCTURE AND DISTRIBUTION OF A2AR

A2AR is a typical G protein‐coupled receptor, which is mainly coupled to Gs protein, containing 7 transmembrane regions, and its C‐terminus is intracellular while the N‐terminus is extracellular.^11^ A2AR is encoded by the Adora2a gene and is located at 22q11.2 chromosome. The A2A receptor gene structure is highly conserved in mice and humans. The protein structure of A2AR consists of 410 amino acids, and these amino acid sequences are highly homologous in mammals.^12^, ^13^ A2AR is considered to be one of the best structurally characterized G protein‐coupled receptors (GPCRs), and more than 30 structures have been deposited in protein databases. These structures exist in three forms: inactive, intermediate active, and active conformational states.^14^ A2AR is highly expressed in tissues such as liver, spleen, thymus, and dopaminergic regions of the brain, and is also expressed in the heart, lungs, and so forth.^15^, ^16^ On cell types, the A2ARs are expressed on most immune cells and platelets.^17^

## 3 ADENOSINE PRODUCTION PATHWAY

**Figure 1:**
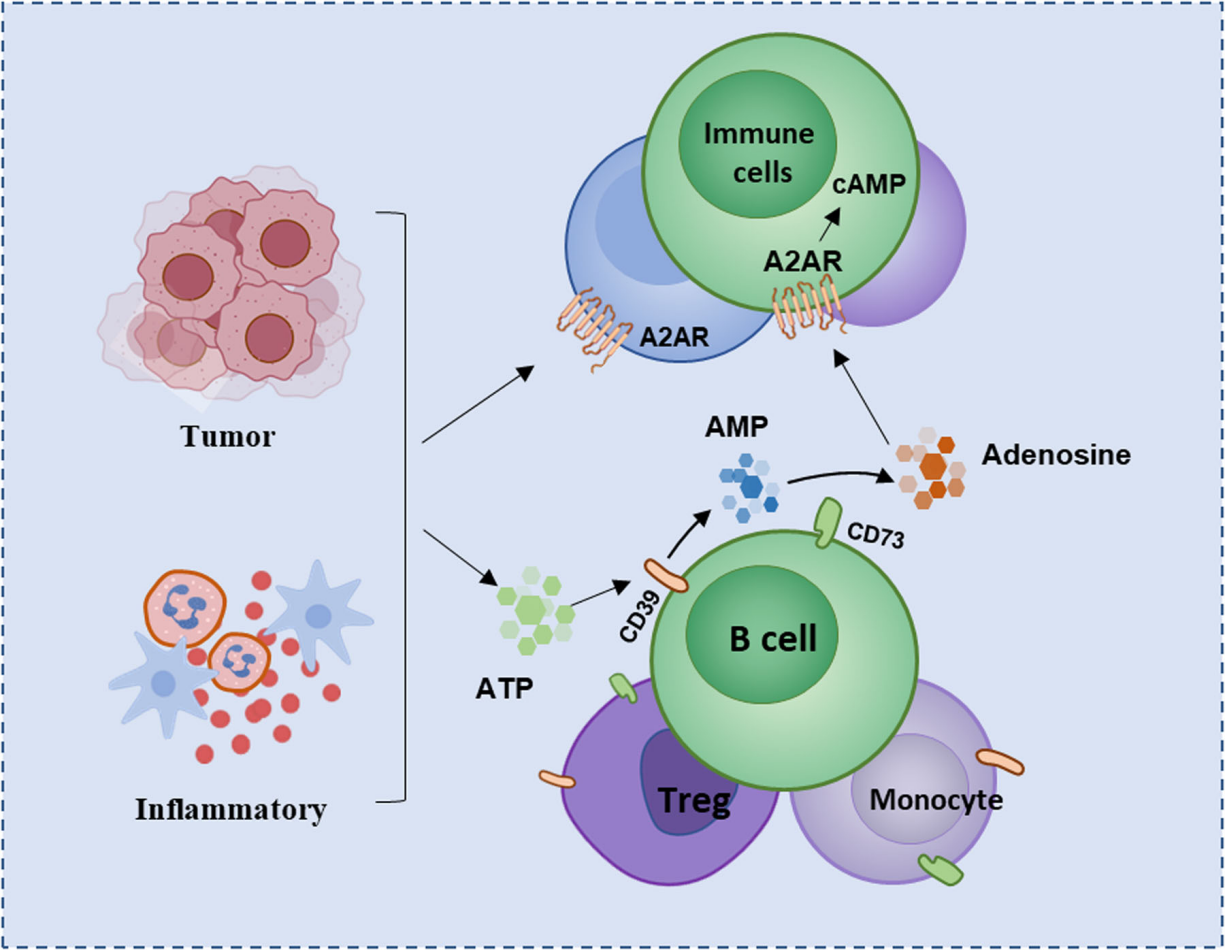
The production pathway of adenosine and the expression of A2AR. Tissues or cells produce high concentrations of ATP under conditions such as hypoxia and inflammation. CD39 and CD73 are highly expressed on immune cells, and ATP produces adenosine under the catalysis of CD39 and CD73. ATP, adenosine triphosphate.

A2AR expressed on the surface of immune cells binds to adenosine can activate downstream signaling pathways and play an important role in immune regulation. Adenosine is mainly produced by the metabolism of extracellular adenosine triphosphate (ATP).^18^ The extracellular adenosine and ATP in normal tissues maintain a low concentration under physiological conditions, but various cells in the tissues can rapidly release ATP to extracellular in some pathological conditions, such as ischemia, inflammation, or hypoxia in the body tissue, resulting in high concentrations of extracellular ATP.^19^ ATP is then hydrolyzed to adenosine by a cascade of two exonucleases, CD39 and CD73.^20^ CD39 is a calcium and magnesium ion‐dependent extracellular nucleotide hydrolase, mainly expressed on the cell membrane of endothelial cells and immune cells, and highly expressed in B lymphocytes and monocytes.^21^ CD73 is a multifunctional glycoprotein anchored outside the cell membrane through glycosyl‐phosphatidylinositol. In some cell types such as regulatory T cells (Tregs) and most B cells, CD39 and CD73 can be co‐expressed on the cell surface.^22^, ^23^ The accumulated ATP is converted into adenosine monophosphate (AMP) under dephosphorylation of CD39, while AMP is dephosphorylated into adenosine catalyzed by CD73, thereby increasing the concentration of extracellular adenosine^24^ (Figure 1). In addition to this classical pathway, two other pathways exist for production of adenosine. NADH is converted into AMP under catalysis of CD38 and CD203a, and AMP is further catalyzed by CD73 to generate adenosine; Another nonclassical pathway for adenosine production is hydrolysis of S‐denosylhomocysteine (SAH) by S‐adenosylhomocysteine (SAHH).^25^

## 4 ADENOSINE‐A2AR SIGNALING PATHWAY

**Figure 2:**
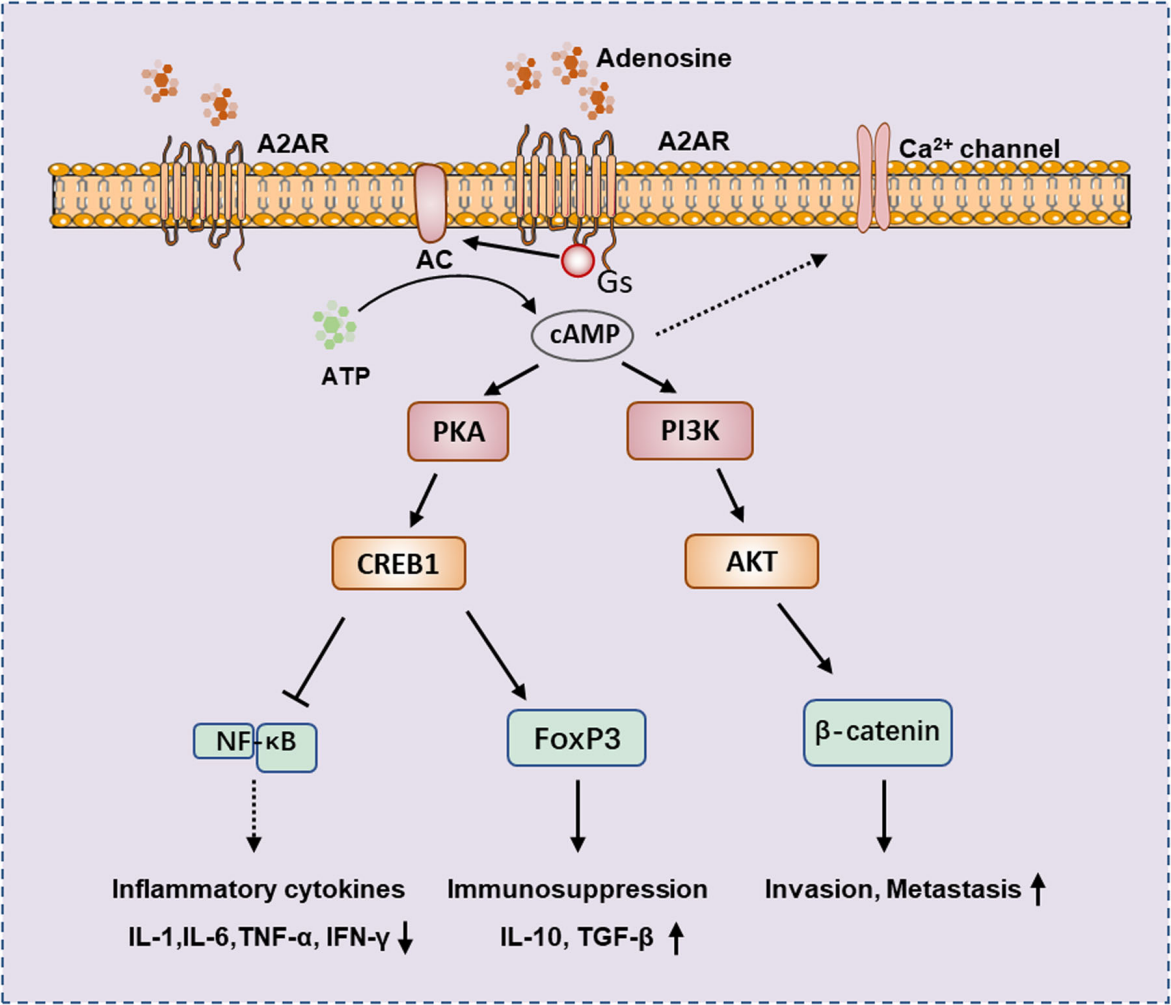
Adenosine/A2AR signaling pathways in immune regulation. The combination of adenosine and A2AR generates cAMP, which further activates PKA, PI3K, and Ca^2+^ channel, causing a series of immune responses.

The A2A receptor binds to adenosine or other agonists, and through the coupled Gs protein, activates adenylate cyclase (AC), which converts ATP to cAMP. The increased cAMP will further activate downstream signaling pathways and affect various biological effects such as tumor development, inflammatory response, and immune tolerance. cAMP can activate protein kinase A (PKA), and leads to cAMP response element‐binding protein (CREB) Ser‐133 phosphorylation and activation, the activation of CREB can inhibit NF‐κB signaling pathway, thereby preventing pro‐inflammatory cytokines secretion. Activation of CREB can also induce the expression of forkhead box protein 3 (Foxp3) and inhibitory cytokines IL‐10 and TGF‐β, thereby affecting differentiation of Treg.^26^, ^27^ Elevation of cAMP can affect tumor invasion and metastasis by affecting PI3K/AKT and Wnt/β‐Catenin pathways.^28^, ^29^ In addition, elevated cAMP also inhibits Ca^2+^ channels, which are crucial for activating lymphocytes, monocytes, neutrophils, mast cells, and other cells.^30^ Conversely, the promoter region of the A2AR gene has binding sites for NF‐κB and signal transducerand activator of transcription 1 (STAT1), which are activated by transcription factors that induce the A2AR expression, thus forming a complex network of regulatory interactions and feedback regulation^31^, ^32^ (Figure 2).

## 5 REGULATION OF A2AR RECEPTORS ON IMMUNE CELLS

A2AR can be expressed on most immune cells, and its expression is complexly regulated by different cytokines. IL‐1 and TNF can increase the expression and function of A2AR on monocytes, among which TNF prevents A2AR desensitization by regulating the binding of GPCR kinase‐2 (GRK2) to the cell membrane, thereby affecting the expression and function of A2AR.^33^, ^34^ Another Th1‐type cytokine, IFN‐γ, downregulated the expression and function of A2AR.^35^, ^36^ On the other hand, changes in the A2AR expression and activation of signaling pathways can also affect the functions of various immune cells. A2AR agonists cooperate with TLR ligands switch pro‐inflammatory M1‐like macrophages towards anti‐inflammatory M2‐like macrophages, resulting in high expression of IL‐10 and low expression of IL‐12 and TNF.^37^ Adenosine/A2AR pathway can also inhibit the antigen‐presenting ability of dendritic cells (DCs) and promote production of immunosuppressive molecules by DCs, such as IL‐10 and indoleamine‐2,3‐dioxygenase (IDO).^38^ Activation of A2AR can significantly reduce the killing activity of natural killer (NK) cells, reducing secretion of cytokines, such as IFN‐γ, and inhibiting stimulation of IL‐12 and IL‐18 on NK cells. The selective inhibitors of the A2AR pathway can reverse these effects.^39^ Activation of A2AR by adenosine inhibits IL‐2 production in CD4^+^ T cells and causes downregulation of CD28 expression on T cells.^40^ Activation of the A2AR pathway also inhibits the proliferation, differentiation, and maturation of CD8^+^ T cells, as well as production of cytokines, such as INF‐γ and TNF.^41^, ^42^ In addition to suppressing the maturation and function of immune effector cells, the adenosine/A2AR pathway can also induce the production of immunosuppressive cells. Activated A2AR promotes transformation of more naive T cells into immunosuppressive Tregs through the Foxp3 and lymphocyte activating gene 3 (LAG3) pathways.^43^ Triggering activation of the A2AR signaling pathway also increases the frequency of myeloid‐derived suppressor cells (MDSCs) in the tumor microenvironment.^44^ Studies have also shown that activation of the A2AR signaling pathway on T cells can also increase the expression of inhibitory receptors involved in immune exhaustion, such as programmed cell death protein 1 (PD‐1), T cell immunoglobulin domain and mucin domain‐3 (Tim‐3).^45^, ^46^ In conclusion, the A2AR inhibits the function of effector immune cells and enhances the activity of immunosuppressive cells, thereby preventing excessive inflammatory responses, protecting organs from damage, and promoting tissue repair. However, once this immune regulation is unbalanced, it may promote immune escape of cancer, or promote the occurrence of autoimmune diseases.

## 6 THE ROLE OF A2AR RECEPTOR IN CANCERS AND AUTOIMMUNE DISEASES

### 6.1 A2AR and cancers

There are ischemia, hypoxia, and inflammatory responses in the tumor microenvironment, which lead to accumulation of large amounts of adenosine in tissues, and the upregulation of A2AR receptor expression can also occur under hypoxic conditions.^47^ Adenosine/A2aR pathway induces a broad immunosuppressive response that modulates uncontrolled inflammation and deleterious injury. The adenosine/A2AR‐mediated pathway may be hijacked to promote immune escape, which in turn leads to tumor progression. Table 1 provides a comprehensive summary of the expression and functional significance of A2AR in both cancers and autoimmune diseases.

**Table 1:** Overview of the involvement of A2AR in cancers and autoimmune diseases.

	A2AR expression	Major finding	Reference
Cancers			
Colorectal cancer	Highly expressed in tumor tissues	High expressions of A2aR and PD‐L1 were associated with a poor prognosis of colorectal cancer	[48, 49]
Gastric cancer	Up‐regulation on gastric cancer tumor tissue and CD8^+^ T cells	A2AR expression was associated with TNM stage, lymph node metastasis, distant metastasis and poor prognosis	[50, 51]
Renal cell carcinoma	Up‐regulated in tumor tissue	A2AR expression was associated with renal cell carcinoma metastasis, resistance to immune‐targeted therapy, and shortened OS	[52, 53]
Breast cancer	Up‐regulated in the cancer tissues	The activation of A2AR increased the proliferation and invasion ability of breast cancer cells, and was associated with tumor growth and metastasis	[29]
Melanoma	The expression levels of A2aR and A2BR are higher than those of A1AR and A3AR in human melanoma cell lines	Adenosine can enhance the proliferation of melanoma cells through A2AR, and on the other hand, the activation of A2AR may also induce cell death	[54, 55]
Autoimmune diseases			
Rheumatoid arthritis	Up‐regulated in peripheral leukocytes in RA patients	Activation of A2AR inhibits NF‐κB signaling pathway and IL‐1, IL‐6 and TNF production	[56]
Ankylosing spondylitis	Up‐regulated on lymphocytes and monocyte‐derived macrophages in AS patients	A2AR activation inhibits the activation of NF‐κB and suppressed TNF, IL‐1β, IL‐6, MMP‐1, MMP‐3 production but increased IL‐23 mRNA expression	[56, 57]
Systemic lupus erythematosus	Higher levels of A2AR expression were found on T cells in SLE patients	Activation of A2AR inhibits inflammatory cytokines (IFN‐α, TNF, IL‐2, IL‐6, IL‐1β) and increases the anti‐inflammatory cytokine IL‐10 release	[58, 59]
Inflammatory bowel diseases	A2AR mRNA was increased in the colonic mucosal tissue of patients with active Crohn's disease	A2AR activation reduce inflammatory cytokine release, decreased the infiltration of lymphocytes, thereby reduced intestinal mucosal inflammation	[60, 61]
Type 1 diabetes	Increased in coronary smooth muscle and endothelial cells in type I diabetic mice	Activation of A2AR regulates coronary blood flow, inhibits NETosis, and exerts a protective effect in a T1DM model	[62, 63]
Multiple sclerosis	Up‐regulated in lymphocytes of MS patients	Inconsistent roles of A2AR appear in the EAE models	[64, 65, 66]

Abbreviations: EAE, experimental autoimmune encephalomyelitis; TNM, tumor node metastasis.

The Caco‐2 cell line is a human colon adenocarcinoma cell model. In vitro cell experiments showed that adenosine binds to A2AR and activates caspase‐9 and downstream molecules, thereby inducing apoptosis of Caco‐2 cells.^48^ A2AR was highly expressed in tumor tissues of colon cancer patients, and was significantly associated with tumor size, depth of invasion and tumor node metastasis (TNM) stage, which could be used as an independent prognostic marker in colorectal cancer.^49^

In gastric cancer tissues, the frequency of A2AR^+^ CD8^+^ T cells was elevated and significantly correlated with lymph node metastasis, distant metastasis and TNM stage. In vitro co‐culture experiments showed that Tregs promoted the apoptosis of CD8^+^ T cells and inhibited the immune activity of CD8^+^ T cells through the adenosine/A2AR pathway.^50^ A2AR expression was increased in cell lines and gastric cancer tissues and was associated with poor prognosis. Further studies showed that the PI3K‐AKT signaling pathway mediates invasion and metastasis of gastric cancer cells.^51^ Adenosine/A2AR can also enhance GC cell stemness by activating the PI3K/AKT/mTOR pathway, leading to radioresistance.^67^

A2AR expression was also up‐regulated in tumor tissues of patients with renal cell carcinoma and was significantly associated with renal cell carcinoma metastasis, resistance to immune‐targeted therapy, and shortened overall survival (OS).^52^ A phase 1 clinical trial demonstrated that a small molecule antagonist of the A2AR can safely block adenosine signaling and promotes tumor regression and improve patient survival in refractory renal cell carcinoma.^53^

The expression of A2AR was significantly up‐regulated in the cancer tissues from breast cancer patients. Experiments in mouse models showed that adenosine can participate in the invasion and metastasis of breast cancer by activating the AKT‐β‐catenin pathway after binding to A2AR.^29^ Nanoparticle‐loaded A2AR siRNA can silence the expression of A2AR on T cells in breast cancer cells and mouse models, and enhances dendritic cell‐based immunotherapy.^68^

Melanoma is one of the most aggressive cancers, caused by melanocytes present in the skin or mucous membranes.^54^ There are limited studies on expression of A2AR in human melanoma tissues, but studies have shown that the mRNA levels of A2AR and A2BR are higher than those of A1AR and A3AR in human melanoma cell lines.^55^ The role of A2AR in melanoma cell lines is complex. Adenosine can enhance proliferation of melanoma cells through A2AR, and on the other hand, the activation of A2AR may also induce cell death.^69^

### 6.2 A2AR and autoimmune diseases

Autoimmune diseases refer to a class of diseases caused by the body's immune response to self‐antigens. It has various clinical manifestations and seriously endangers human health, but its etiology is still unclear. There are multiple mechanisms for regulation of A2AR in autoimmunity. Adenosine/A2AR signaling pathway can affect the production of inflammatory cytokines and chemokines in innate immune cells.^70^ It can also affect adaptive immunity and regulate the function of Treg, Th17, Th1, Th2, and regulatory B‐cells (Breg cells).^71^, ^72^ The adenosine/A2AR pathway can also affect the formation of neutrophil extracellular traps (NETs), which have a role in suppressing the spread of pathogens and are also associated with autoimmune diseases. Activation of A2AR reduces the formation of NETs, thereby downregulating the pro‐inflammatory effects of NETs and preventing development of autoimmunity.^73^

Rheumatoid arthritis (RA) is an autoimmune disease characterized by chronic, erosive polyarthritis. It is mainly manifested as symmetrical, persistent and progressive polyarthritis involving small joints such as hands, arms and feet. Methotrexate is the drug of choice for patients with rheumatoid arthritis. Research as early as 1991 showed that methotrexate promotes adenosine release, thereby reducing the inflammatory response.^74^ Subsequent experiments demonstrated that adenosine mediates anti‐inflammatory effects of methotrexate by binding to A2a and A3.^75^, ^76^ A2AR expression is upregulated in leukocytes of RA patients and inhibits the NF‐κB signaling pathway by reducing TNF, IL‐1, and IL‐6.^56^

Ankylosing spondylitis (AS) is a chronic, progressive, and inflammatory autoimmune disease that mainly invades the sacroiliac joints and central axis of the spine, and can also involve peripheral joints and tendon attachments to varying degrees. The expression of A2AR was up‐regulated on lymphocytes of patients with ankylosing spondylitis in another study, and agonists of A2AR inhibited the activation of NF‐κB and production of TNF, IL‐1β, IL‐6, MMP‐1, and MMP‐3.^56^ Compared with healthy controls, monocyte‐derived macrophages from AS patients expressed elevated mRNA level of A2AR and were negatively correlated with AS activity scores.^77^ A2AR expression was also upregulated in monocyte‐derived macrophages from AS patients, and activation of A2AR suppressed TNF production but increased IL‐23 mRNA expression in macrophages.^57^ A2AR has also been identified as a promising therapeutic target in mouse models of autoimmune arthritis. Activation of the A2AR signaling pathway can inhibit CD4 germinal center (GC)‐follicular helper T (Tfh) differentiation, thereby reducing differentiation of autoreactive B cells that promote arthritis.^78^ A2AR is expressed on most inflammatory cells, indicating that A2AR has great potential as a therapeutic target for rheumatoid arthritis and inflammatory diseases.

Systemic lupus erythematosus (SLE) is a typical autoimmune disease that produces a large number of autoantibodies and involves multiple organs. The etiology of SLE has not yet been fully clarified, and studies have shown that the immune system dysfunction is related to pathogenesis of this disease.^79^, ^80^ Higher levels of A2AR expression were found on T cells in SLE patients compared to healthy controls and were inversely correlated with disease activity.^58^ Moreover, activation of A2AR reduces the NF‐κB signaling and diminishes production of inflammatory cytokines (IL‐1β, IL‐6, IFN‐α, TNF), increasing the anti‐inflammatory cytokine IL‐10 release.^59^ Studies in mice also confirmed the protective function of the A2A receptor in SLE, as treatment of MRL/lpr mice with A2AR agonist CGS21680 inhibited T cell activation, autoantibody production, and renal injury.^81^ This suggests that, the A2AR is involved in the regulation of complex pathogenesis of SLE, and potential application of A2AR agonists in the treatment of SLE.

Inflammatory bowel disease (IBDs) are a class of autoimmune gastrointestinal diseases, including ulcerative colitis and Crohn's disease, characterized by a dysregulated immune response against antigens of bacterial origin that occurs within the intestinal mucosa.^60^ The mRNA expression of A2A receptors was increased in the colonic mucosal tissue of patients with active Crohn's disease. The expression of A2AR is not significantly changed in ulcerative colitis patients.^61^ ATL‐146e is a selective agonist of A2AR, that can inhibit the release of pro‐inflammatory cytokines and decrease the infiltration of lymphocytes, thereby reducing intestinal mucosal inflammation.^82^ Therefore, selective agonists of A2AR are promising as a new strategy for the treatment of inflammatory bowel disease.

Type 1 diabetes is an autoimmune disease characterized by progressive damage to pancreatic islet B cells and insufficient insulin secretion. Experimental evidence from mouse models has suggested that, CD39 overexpression provides protection in a multiple low‐dose streptozotocin‐induced model of diabetes by activating adenosine 2A receptor receptors.^62^ A2AR expression was significantly increased in coronary smooth muscle and endothelial cells in a mouse model of type 1 diabetes mellitus (T1DM), and activation of A2AR plays key role in the regulation of coronary blood flow.^63^ A2AR can suppress NETosis in a low‐dose streptozotocin‐induced T1DM model, and plays a protective role in reducing tissue damage.^83^ A2AR gene variants have also been shown to be protective against retinopathy in type 1 diabetes.^84^

Multiple sclerosis (MS) is a chronic autoimmune disease involving the central nervous system, and experimental autoimmune encephalomyelitis (EAE) is the most widely used research model of MS.^85^ Consistent roles of A2AR appear in the EAE models. Genetic deletion of A2AR produces more inflammatory cell infiltration and a more severe demyelinating phenotype.^86^ A2AR upregulation was observed in lymphocytes of MS patients compared with healthy controls, and activation of A2AR receptors mediated inhibition of NF‐κB pathway and inhibition of pro‐inflammatory cytokine production.^64^ This also suggests that the A2AR agonists could serve as a new MS therapeutic tool. But there are also studies that suggest the opposite. A2AR antagonists significantly reduced neuroinflammation, including reduced inflammatory cell infiltration, spinal cord macrophage numbers, and demyelination, have a protective effect on EAE.^65^ The study by Mills et al.^66^ had similar findings. This contradiction reflects the complex role of A2A receptors in regulating EAE pathology. In the EAE model, the A2AR signaling pathway may play different functions in immune cells and nonimmune cells.

## 7 PHARMACOLOGICAL TARGETING OF A2AR SIGNALING

Based on the pharmacological properties of A2AR, several methods targeting A2AR receptors and their signaling pathways have been studied and developed. Currently, the main therapeutic methods targeting the A2AR signaling pathway include the use of A2AR agonists or antagonists, which have been extensively researched^87^, ^88^; indirectly targeting the receptor by regulating adenosine concentration,^89^ which is a method with fewer side effects; the use of prodrugs, which is a more tissue‐targeted method, with some studies using prodrugs that are selectively cleaved by CD73 in inflammatory tissue to act as A2AR agonists^90^; and the use of synthetic drugs that can target multiple receptors, such as those with dual action of A2AR agonist and adenosine transporter inhibitor, which have shown efficacy in neurodegenerative diseases.^91^ Gene knockout of A2AR is also an effective method for targeting the A2AR signaling pathway.^92^ In addition, allosteric modulation of A2a receptors is a new therapeutic approach, with these allosteric modulators potentially having more therapeutic advantages than classical agonist and antagonist molecules.^93^ Therefore, the development of efficient and targeted A2a receptor drugs and methods, including allosteric modulators and gene knockout, will become an important direction for future treatments of diseases such as tumors, autoimmune diseases, and neurodegenerative diseases.

## 8 TARGETED THERAPIES OF A2AR SIGNALING

Based on critical immunomodulatory role of the adenosine/A2AR pathway in tumor immune escape and autoimmunity, the adenosine/A2AR pathway is a novel target for treatment of cancer and autoimmune diseases. For cancers, since the hypoxia‐adenosine axis is more prominent in the tumor microenvironment, targeting the A2AR may have a better safety profile than other immunotherapeutic approaches. At present, some A2AR inhibitors have entered the clinical trial stage, such as CPI‐444, AZD4635, AB928, PBF‐509, and so forth. The intervention strategies in these clinical trials include monotherapy, combined with PD‐L1 and CD73 antibody, and so forth.^94^ Targeting multiple targets for the adenosine pathway using a combinatorial strategy has been validated in preclinical models, and dual blockade can inhibit spontaneous lung metastasis and more effectively enhance antitumor immunity.^8^ For renal cell carcinoma, trials using the A2AR antagonist CPI‐444 as monotherapy and in combination with the PD‐L1 inhibitor atezolizumab have yielded positive results, with median progression‐free survival (PFS) 4.1 months and 5.8 months, respectively.^53^ CRISPR/Cas9‐mediated deletion of A2AR in mouse and human‐derived chimeric antigen receptor T‐Cells (CAR T‐cells) abolished the immunosuppressive effect of adenosine, and targeting A2AR by this strategy significantly enhanced CAR T‐cell efficacy, resulting in improved mouse survival.^92^ The combined use of A2AR inhibitors and CAR T therapy can greatly increase the antitumor effect, which also increases application scope and prospects of A2AR antagonists. Activation of the A2AR signaling pathway by selective agonists also represents a new anti‐inflammatory approach with potential in the treatment of autoimmune diseases. A nonabsorbable, locally active A2AR agonist, 4‐(2‐ethyl)‐benzenesulfonic acid (7, PSB‐0777), has shown encouraging results and was recently proposed as a promising candidate for the treatment of inflammatory bowel syndrome.^95^

## 9 CONCLUSIONS

A2AR receptor is a high‐affinity receptor involved in immune regulation among the adenosine receptors. The hypoxic environment in the local tumor or external stimuli converts ATP into adenosine, and adenosine activates A2AR to play an immunosuppressive function through the cAMP pathway. A2AR is expressed in most immune cells and can regulate differentiation, maturation and function of immune cells. The role of A2AR on immune regulation is extensive, and it can regulate the functions of various immune cells, participating in both innate and adaptive immune responses. A2AR also has complex effects on immune regulation, and activation of A2AR pathway can inhibit the production and release of inflammatory cytokines. This immunosuppressive effect plays a protective role in autoimmune diseases. However, this immunosuppressive effect allows tumor cells to escape clearance by the immune system. Therefore, when using the A2AR agonists or antagonists, it is necessary to clarify the specific role of A2AR in the corresponding diseases to obtain effective immune protection.

## AUTHOR CONTRIBUTIONS

Hongling Ye wrote the manuscript draft. Junqi Zhao, Xuejing Xu and Dagan Zhang, contributed to the gathering of data. Junqi Zhao did the editing. Han Shen and Sen Wang helped to organize and revise the manuscript. And Han Shen and Sen Wang were the funding recipients. All authors have read and approved the final manuscript.

## CONFLICT OF INTEREST STATEMENT

The authors declare no conflict of interest.

## Data Availability

All the information included in this manuscript is available upon request by contact with the corresponding author.
